# Guiding Principles for Adolescent Web-Based Portal Access Policies: Interviews With Informatics Administrators

**DOI:** 10.2196/49177

**Published:** 2024-03-11

**Authors:** Bryan Sisk, Alison L Antes, Christine Bereitschaft, Madi Enloe, Fabienne Bourgeois, James DuBois

**Affiliations:** 1 Bioethics Research Center Department of Medicine Washington University School of Medicine St Louis, MO United States; 2 Division of Hematology and Oncology Department of Pediatrics Washington University St Louis, MO United States; 3 Boston Children's Hospital Boston, MA United States; 4 Harvard Medical School Boston, MA United States

**Keywords:** adolescent, patient portal, electronic health records, policy, ethics, portal, portals, adolescents, youth, health record, health records, EHR, EHRs, perspective, perspectives, policies, administrator, administration, informatics, information system, information systems, guidelines

## Abstract

**Background:**

Web-based patient portals are tools that could support adolescents in managing their health and developing autonomy. However, informatics administrators must navigate competing interests when developing portal access policies for adolescents and their parents.

**Objective:**

We aimed to assess the perspectives of informatics administrators on guiding principles for the development of web-based health care portal access policies in adolescent health care.

**Methods:**

We interviewed informatics administrators from US hospitals with ≥50 dedicated pediatric beds. We performed a thematic analysis of guiding principles for developing and implementing adolescent portal access policies.

**Results:**

We interviewed 65 informatics leaders who represented 63 pediatric hospitals, 58 health care systems, 29 states, and 14,379 pediatric hospital beds. Participants described 9 guiding principles related to three overarching themes: (1) balancing confidentiality and other care needs, (2) balancing simplicity and granularity, and (3) collaborating and advocating. Participants described the central importance of prioritizing the health and safety of the adolescent while also complying with state and federal laws. However, there were differing beliefs about how to prioritize health and safety and what role parents should play in supporting the adolescent’s health care. Participants also identified areas where clinicians and institutions can advocate for adolescents, especially with electronic health record vendors and legislators.

**Conclusions:**

Informatics administrators provided guiding principles for adolescent portal access policies that aimed to balance the competing needs of adolescent confidentiality and the usefulness of the portal. Portal access policies must prioritize the adolescent’s health and safety while complying with state and federal laws. However, institutions must determine how to best enact these principles. Institutions and clinicians should strive for consensus on principles to strengthen advocacy efforts with institutional leadership, electronic health record vendors, and lawmakers.

## Introduction

Web-based patient portals are widely available tools that can improve patients’ sense of control [1-4], adherence [1], and medical understanding [2,3,5]. Portals represent an opportunity to engage adolescents in health care and support their developing autonomy. However, adolescents could experience emotional distress or frustration when reviewing results or clinical notes through the portal, especially if they receive difficult news such as a cancer diagnosis. These portals also risk divulging confidential information to parents that an adolescent has shared with a clinician [6,7]. Adolescents are less likely to communicate transparently with clinicians if they have concerns about confidentiality [8,9]. These concerns could lead adolescents to forgo sensitive medical care that could result in serious health repercussions, such as sexually transmitted infections, unplanned pregnancies, or poor mental health. However, parents often play an important role in managing the adolescent’s health, and US public opinion supports parental access to adolescents’ health care records [10]. Additionally, most adolescents rely on their parents’ support to manage or comanage their health care, especially if the adolescent has a chronic illness [11]. To provide ethical and effective access to adolescent portals, institutions must strive for ideal strategies that balance confidentiality and usefulness [12-14].

The 21st Century Cures Act mandates that US health care systems allow patients access to their electronic health record (EHR) data, typically through web-based patient portals [15]. We previously found that pediatric institutions have used widely varying policies for adolescent portal access across the United States [16]. Although most studies of adolescent portal use have been performed in the United States [17], other countries are similarly providing portal access to adolescents and their parents [18,19]. Variations in portal policy are driven, in part, by adolescent confidentiality laws that vary by state [20]. Each state has unique confidentiality laws with categories of protected information that generally include information about reproductive health, substance use, sexually transmitted illnesses, and mental health [21,22]. However, even within states, health care systems have interpreted the same laws differently, leading to different access policies [16]. Similarly, regulations vary in other countries. For example, the General Data Protection Regulation of the European Union requires a patient to be 16 years old to provide digital consent. The 21st Century Cures Act mandate for transparency has encouraged institutions to further reevaluate these adolescent portal access policies and their interpretation of laws.

Few studies have engaged administrators to understand their perspectives on guiding principles for developing and implementing adolescent portal access policies following the 21st Century Cures Act. Several professional medical societies have published guidelines and policy statements about adolescent portal policies and focused mainly on preserving confidentiality [13,14,23,24]. However, it is essential to understand the perspectives of administrators who are charged with developing and implementing these policies because they have rich experiential insights into the challenges of administrating adolescent portal access in the US health care system. In the United States, these administrators often work in teams that include technical staff and clinicians with informatics expertise. These groups also collaborate with risk management and legal counsel to develop adolescent portal access policies that they perceive to be compliant with state and federal laws. We interviewed 65 informatics administrators from multiple health systems across the United States. Our prior analysis of these interviews characterized the varying adolescent portal policies across the United States [16], as well as approaches to engaging adolescents in using the portal [25]. In this analysis, we aimed to identify guiding principles to inform the development of these policies in the future.

## Methods

### Overview

We report these findings following the Standards for Reporting Qualitative Research (SRQR) checklist [26] (Multimedia Appendix 1).

### Participants and Recruitment

We performed structured interviews with informatics administrators who oversaw adolescent portal access policies. Informatics administrators were eligible if they were involved in developing or implementing adolescent portal policies and if they oversaw a US children’s hospital with ≥50 dedicated pediatric beds. Specialty and rehabilitation hospitals were ineligible. We identified children’s hospitals using the Children’s Hospital Association (CHA) database in January 2022. Of 232 children’s hospitals, we excluded specialty or rehabilitation hospitals (n=37), non-US hospitals (n=7), and hospitals with <50 pediatric beds (n=9), yielding 179 eligible hospitals. We recruited participants through 2 email groups of informatics administrators and simultaneously identified contact information for informatics administrators through publicly available data. These email groups included informatics administrators across the United States who opted into the list to communicate with fellow informatics leaders. After initially sending recruitment materials through these email groups and receiving some responses, we then sent targeted emails to administrators at each remaining children’s hospital listed in the CHA database. We emailed administrators at every eligible children’s hospital to request interviews. We also included administrators from US hospitals with which the authors were affiliated, given the importance of capturing representative data across the United States.

### Data Collection

We identified the number of pediatric beds from the CHA database, supplemented with information from hospital websites. We developed a structured interview guide that explored adolescent portal policies, factors influencing the development and implementation of policies, and approaches to engaging adolescents through portals (Multimedia Appendix 2). We specifically asked for advice from other informatics administrators and guiding principles for developing adolescent portal access policies. This interview guide was developed through a literature review and engagement with informaticists. We revised the interview guide with a stakeholder advisory board and 3 informatics administrators. This advisory board included 4 physicians with expertise in informatics, primary care, adolescent medicine, and endocrinology, as well as an adolescent with chronic illness and their parents. In the interview guide, we indicated which questions were essential and which questions could be skipped if insufficient time. However, we were able to ask each pertinent question for the current analysis in every interview. BAS conducted interviews between February and July 2022 via telephone or videoconferencing software. Interviews were audio-recorded and professionally transcribed. Interviews ranged from 12 to 43 minutes.

### Data Analysis

Our overall qualitative analysis adhered to the Total Quality Framework, a comprehensive approach that ensures the accuracy, credibility, analyzability, transparency, and usefulness of qualitative findings [27,28]. We used thematic analysis [29] of guiding principles for developing and implementing adolescent portal policies. BAS and ALA developed the codebook. BAS is a pediatric oncologist, ethicist, and communication researcher with training in qualitative research. ALA is an organizational psychologist and ethics researcher with experience and training in qualitative research. Coding involved multiple iterative steps: (1) read transcripts to familiarize themselves, (2) descriptively coded 5 transcripts to formulate preliminary codes, (3) grouped codes into categories and collapsed categories into representative themes, and (4) refined definitions for themes through 3 cycles of independent coding and consensus meetings. After reviewing 25 transcripts, we reached saturation for representative themes. Using this final codebook, BAS, CB, and ME independently coded all transcripts, using these codebook definitions to ensure consistent and reliable application of codes. These authors then reviewed the other’s application of codes, marked disagreements, and resolved disagreements through discussion. We used Dedoose (SocioCultural Research Consultants) qualitative software.

### Ethical Considerations

The institutional review board at Washington University determined this study was exempt. We obtained verbal informed consent. All transcripts were deidentified prior to analysis.

## Results

### Participant and Health Care System Characteristics

We identified 179 eligible pediatric hospitals and contacted an informatics administrator at every eligible center. We interviewed 65 informatics experts representing 63 hospitals across 58 health care systems. Thus, participants represented 35% of all US children’s hospitals with more than 50 dedicated pediatric beds. EHRs from all participating health systems had web-based health portals in pediatrics. The number of dedicated pediatric beds in participating hospitals ranged from 51 to 664 (median 189, IQR 107-313) beds. In total, participants represented systems with 14,379 dedicated pediatric beds across 29 states plus Washington, District of Columbia (Table 1). The majority of health care systems used Epic EHR systems.

**Table 1:** Characteristics of participants and represented health care systems.

Characteristic			Values
**Professional role of participant (n=65), n (%)**			
	Chief medical information officer	34 (52)	
	Clinical informaticist	15 (23)	
	Chief information officer^a^	3 (5)	
	Other^b^	13 (20)	
**Type of electronic health record (n=58), n (%)**			
	Epic	41 (70)	
	Cerner	9 (16)	
	Multiple	5 (9)	
	Allscripts	1 (2)	
	Other	2 (3)	
**Pediatric-specific informatics team (n=58), n (%)**			
	Yes	31 (53)	
	No	27 (47)	
**Pediatric-specific instance of EHR^c^ (n=58), n (%)**			
	Yes	20 (34)	
	No	38 (66)	
**Number of dedicated pediatric hospital beds (n=58)**			
	Range	51-664	
	Median (IQR)	189 (107-313)	
**Age of adolescent access (years; n=58), n (%)**			
	No access provided	8 (15)	
	10	1 (2)	
	11	2 (3)	
	12	14 (24)	
	13	21 (36)	
	14	7 (12)	
	15	2 (3)	
	16	1 (2)	
	Unsure	2 (3)	
**Are parents permitted proxy access? (n=58), n (%)**			
	Yes	55 (95)	
	No	3 (5)	
**Are adolescents permitted access? (n=58), n (%)**			
	Yes	43 (74)	
	No	8 (14)	
	Unsure	7 (12)	

^a^Includes 1 participant who identified as a director of health information systems.

^b^Other roles included pediatric service line lead, director of nursing informatics, director of quality, certified analyst, adolescent physician, director of clinical analytics, medical director of informatics, chief medical officer, and clinician champion.

^c^EHR: electronic health record.

### Guiding Principles and Advice for Developing Adolescent Portal Access Policies

#### Overview

Participants described 9 guiding principles related to three overarching themes: (1) balancing confidentiality and other care needs, (2) balancing simplicity and granularity, and (3) collaborating and advocating. We describe each of these themes and principles in Table 2 and subsequent sections.

**Table 2:** Subtheme definitions for guiding principles for developing and implementing adolescent portal policies.

Themes and subthemes			Representative excerpts
**Balancing confidentiality and other care needs**			
	Provide appropriate transparency while complying with state and federal laws	Compliance with laws and regulations was noted as an essential requirement for any portal policy. The overarching goal of improving transparency was shared by nearly all administrators, but there were differences in how to achieve this transparency in ethically and legally acceptable ways. Administrators described the vagueness of these laws and several instances with state and federal laws are in conflict. As a result, administrators described the importance of developing a productive, collegial working relationship with the institutional compliance office and legal counsel.	
	Prioritize adolescent health and safety	Administrators strongly believed that all policies should aim to ensure the health and safety of the adolescent. However, health and safety could sometimes be in conflict. For example, some adolescents might need parental involvement to help them manage complex disease. Other adolescents might be unsafe if their parents see sensitive information, such as drug use, gender identity, or sexual activity.	
	Preserve clinician-adolescent relationship	Administrators highlighted the importance of ensuring that portal policies support rather than strain the clinician-adolescent relationship. Clinicians especially needed to honor their promises of confidentiality or they would risk losing the adolescent’s trust.	
	Support adolescent’s developing autonomy	Although administrators had disagreements about what level of access is appropriate or mandated for parents, most administrators expressed that adolescents should have access to as much of their own information as possible. Not only do adolescents have a right to learn about their own health, but administrators believe that this access could support the adolescent’s development into adulthood and self-management.	
**Balancing simplicity and granularity**			
	Strive for appropriate granularity in the differential sharing of health information	Administrators generally agreed that some granularity in the ability to determine which information is shared with parents versus adolescents is ideal. Some described an ideal in which adolescents could determine each type of information that is shared with their parents. However, technological limitations created barriers for differential sharing, especially at centers with smaller pediatric populations.	
	Ensure the end product is useful for families	Administrators urged vendors and institutions to ensure the user interface was user-friendly and provided meaningful information for parents and adolescents. They also described how it was important to make sure information was understandable to families. Simply providing access was not sufficient. Additionally, administrators urged against the view of the portal as a panacea for all communication and information challenges.	
**Collaborating and advocating**			
	Engage key stakeholders within the institution	Administrators emphasized the importance of engaging with leadership, informatics workforce, legal or compliance officers, clinicians, frontline staff, parents, and adolescents in developing and implementing access policies. Additionally, they encouraged the ongoing engagement of these parties after implementation to ensure the system continues to meet all parties’ needs.	
	Collaborate with colleagues at other institutions	Given the uncertainty and vagueness of state and federal laws, administrators encouraged other administrators to communicate with colleagues at other institutions to understand the variety of approaches to adolescent portal access. This collaboration was especially important to understand how other hospitals in the same state were interpreting the laws.	
	Advocate with external parties for adolescent and pediatric issues	Many of the conditions influencing adolescent policies originated outside of the institution, especially state laws, federal policies, and EHR^a^ functionality. It is imperative for health care institutions to advocate with these external parties to support safe and transparent sharing of adolescent medical information through portals.	

^a^EHR: electronic health record.

#### Balancing Confidentiality and Other Care Needs

##### Provide Appropriate Transparency While Complying With State and Federal Laws

State-level confidentiality laws varied widely across states, leading to policies that varied in the amount of authority parents have to access their child’s medical information through the portal. The federal mandate against information blocking did not specify how this mandate applied to adolescent health, and the federal law specifically defers to state laws in these matters. Furthermore, federal and state laws were written vaguely, and some perceived the state and federal laws to be in conflict. For a detailed analysis of state-by-state variability, see Sharko et al [22]:

It was a lesson learned how really poorly written state laws are. Whether it’s state or federal or regulations, you think it would spell out exactly what you need to do, but it’s not that way at all. That 1,250-page behemoth that [Office of the National Coordinator for Health Information Technology] produced only muddied the waters even further. We really don’t know what they’re really expecting and they’re not.

Participant #181, chief medical information officer

Participants described how adhering to state laws was a critical foundation for any portal access policy for adolescents:

We have an affirmative requirement to protect certain information and not violate the state laws of [our state]. That is, obviously, something we take extremely seriously.

Participant #176, chief medical information officer

Given the complexity, variation, and vagueness of state laws, participants expressed the essential role of institutional legal and compliance officials:

There are many regulations that are conflicting, and it’s really important to ensure that you are looping in your compliance and legal folks...because ultimately, there’s just laws that are in conflict.

Participant #26, chief information officer

However, the recommended approaches to complying with confidentiality laws varied. While participants described the need to balance transparency and confidentiality, some participants emphasized the importance of prioritizing the adolescent’s confidentiality over transparency and limiting access for both parents and adolescents because this was the “path of least resistance” (participant #78, clinical informaticist), technically easier, and satisfied the concerns of legal and compliance administrators. Additionally, some participants expressed their beliefs that state laws provided protection if they opted to restrict information from both parents and adolescents:

We have not increased access for adolescents...We would defend it based on state laws about confidentiality. If there’s state laws that supersede some of the Cares Act, we can seek protection behind those.

Participant #78, clinical informaticist

Conversely, others emphasized the importance of transparency:

I think that the default assumption should be that teens can access all their own information, and that parents can access all of their kids’ information, except that that’s protected by adolescent health laws.

Participant #153, chief medical information officer

Some participants also described the “importance of parents knowing what’s going on with their [adolescent]” (participant #93, chief medical information officer).

We can’t disconnect the parents from the non-confidential information. I think it’s so important and key for them to be able to continue to meaningfully provide care and safely provide the care that’s required for their adolescent. Disconnecting them from that information, I think, is just the wrong thing to do.

Participant #172, chief medical information officer

However, this point was discussed less frequently than the importance of preserving transparency. Some participants recommended that adolescents should be empowered to determine this balance of transparency and privacy by determining what level of access their parents are permitted: “I think having it be in the adolescent’s hands to determine what their parents have access to and to be the ones in control of that I think is the right approach” (participant #119, chief medical information officer). Participants in other states, however, explained that such an approach might conflict with their state laws that provided parents with rights to access these data.

##### Prioritize Adolescent Health and Safety

In addition to adhering to the law, participants described how the adolescent’s health and safety must be the other central guiding principle for portal access policies: “To me, the guiding principle is always what’s the safety of the patient and what’s in their best interest” (participant #26, chief information officer). Furthermore, some participants described the need to prioritize the adolescent’s health needs over the legal concerns of the institution:

Focus on the patient, not on the lawyers. If we can, again, try and stay focused on what is gonna help us take care of the patients, why do we want to be transparent with this information, understand that nobody is out to get us...Frankly, the government doesn’t have the resources to do any kind of investigation anyway.

Participant #112, chief medical information officer

However, there were differing beliefs about how to prioritize health and safety. For some participants, supporting health requires transparent disclosure of high-quality information to both adolescents and their parents.

Information is powerful. Information helps improve communication, helps improve health outcomes, helps improve quality.

Participant #57, chief medical officer

Additionally, some participants reiterated the need to incorporate parents in the adolescent’s health care, especially for adolescents with serious illness:

I do think for other health conditions, we want to be careful not to set barriers to where the parents can be helpful in helping that adolescent manage those conditions. It's very much a balancing act.

Participant #176, chief medical information officer

Others, however, described how limiting parental access might be necessary to ensure the adolescent’s safety, for example, if they were at risk of abuse from parents following disclosure of sensitive information:

At the very top of the pyramid is patient. All of our decisions, we try to keep that in mind. That’s where, even though I may get frustrated that sometimes there’s access that’s decreased for my parents, if it means that it’s providing the actual patient a little bit more security and privacy, then I’m able to appreciate that this is really what’s best for them.

Participant #38, chief medical information officer

Still, others were uncertain about how to determine what portal policy is best for adolescents:

Yeah. I think it should be about patient—what’s best for the patient. I think that should be—to me, it’s pretty simple. Now, that’s a complex part, right? How much information do you divulge? What do they keep from their parents? What’s the right thing to do? The more ethical issues there, which I don’t have an answer to but, I think, at the end of the day, what’s gonna promote the best health for child and adult?

Participant #140, chief medical information officer

##### Preserve Clinician-Adolescent Relationship

Participants advised clinicians to recognize that adolescents have a right to their own relationship with their clinician:

At the end of the day, we wanna protect their information and their relationship with their provider...I struggle because, as a parent, I want to have access to my child’s information, but I also realize it’s their relationship as well, so, I guess, just protecting their—I don’t know—right to have that relationship with a provider.

Participant #167, chief medical information officer

The trust established in this relationship is essential to engaging adolescents in their care and bolstering the long-term clinical relationship: “I think if [trust is] fractured, then it’s difficult to have an ongoing good relationship with that teenager” (participant #20, chief medical information officer). This trust relies on clinicians honoring their promises of confidentiality:

If we tell them that a conversation is private, it truly is private and that we honor that, and that there is a mechanism for that to truly be information that we do not share without their consent. Otherwise, they’re just never going to trust us. They’re not gonna trust giving us that information or really feeling comfortable engaging with a portal.

Participant #172, chief medical information officer

##### Support Adolescent’s Developing Autonomy

Supporting and developing the adolescent’s autonomy were also goals of many participants:

Patient access to patient portals has a lot of positives, and I think one that gets overlooked is patients taking ownership of their own health care because the portal allows them to learn about themselves at an earlier age, learn what their diagnoses are, what their medications are, who their providers are...I think it’ll help patients understand more about themselves, communicate better with health care professionals, and make them an active participant in their health care.

Participant #57, chief medical officer

They viewed portals as a teaching tool to support the independence of the adolescents:

The portal, I think for the adolescent group, is a way to increase engagement and to, hopefully, teach some of these skills that are going to be lifelong skills. This is like a really pivotal time, and I think we’re missing the opportunity, from that perspective.

Participant #10, clinical informaticist

However, the role of the adolescent must be adapted to their level of development and interest:

We can’t expect a 14-year-old to manage their Type I Diabetes or their own Inflammatory Bowel Disease, but I do think that by giving them access, it does kind of help them take that next step in owning the management of their current diseases.

Participant #138, clinical informaticist

In addition, policies should not force responsibilities on the adolescent if they are not ready or willing to manage their health:

For adolescents that truly want to manage their own health care and want to be engaged to that degree, then they should be the primary user of the portal, with the parent being in a supporting role. On the flip side, if you have a parent and a child relationship where the parent really is managing everything, then they need to retain that.

Participant #181, chief medical information officer

Some participants described the importance of guidance and guardrails to ensure adolescents remain safe. For some participants, the ideal guardrail is comanagement of care between the parent and adolescent, with graduated responsibility for the adolescent over time. Without this support, adolescents might be unable to sufficiently manage their health care:

Is a 13-year-old ready to make their own medical decisions? There’s probably a handful who are, but there’s probably a lot more who struggle with that. I know certainly my kids at 13 wouldn’t have been able to manage their own care.

Participant #133, chief medical information officer

#### Balancing Simplicity and Granularity

##### Strive for Appropriate Granularity in Differential Sharing of Health Information

Many participants described the need for technological advancements that will permit differential sharing of information between the parent and adolescent:

Technology needs to evolve so that parents can be engaged, and teens have the ability to actively, through portals, decide what they’re gonna share and not share because every relationship between a teen and their parents is different and can change on a moment’s notice.

Participant #176, chief medical information officer

The adolescent would ideally control this access, perhaps through widgets on their portal that do not require clinician actions:

I would put the widgets for access right on the portal for the adolescent to control in addition to reupping having an active process for re-upping. I would also make it more autonomous that they can manage the access independently. They don’t have to go through us.

Participant #60, chief medical information officer

Some participants believed that adolescents with complex needs might need different or modified privacy settings to ensure that the child’s medical problems are sufficiently managed:

I think that we try to make it as simple as possible, and this is a rather complex issue. I think that we probably need another type of access for those patients with chronic medical care needs, or if we could pick and choose more easily which things a child was letting their parent see, I think that would make it a little bit easier, and I think I would be more satisfied with it.

Participant #38, chief medical information officer

With this granular sharing, however, some participants worried that allowing the adolescent to censor certain health information might be considered information blocking or might conflict with state laws that consider the medical record to be the parent’s property: “When you cross over into that world where you’re now blocking certain elements from the parent, then you possibly fall into information blocking” (participant #98, clinical informaticist). Another participant further elaborated:

I think that to be fully compliant with the Cures Act and the need to prevent information blocking, we should really only be selectively not sharing that information with the proxy, the third party [and adolescents should retain access to this information]. Right now, at least in our system, we only really have the ability to either have it appear in the portal or not appear in the portal [for both the adolescent and proxy]...That seems unfair to adolescents ‘cause those may be the things they most care about.

Participant #119, chief medical information officer

Conversely, other participants worried that more granular sharing was required to comply with the Cures Act because many health care systems were withholding information from adolescents. To achieve this granularity, a participant encouraged other administrators to “figure out your needs, and then design backwards from that” (participant #52, chief medical information officer).

##### Ensure End Product Is Useful for Families

In addition to the focus on portal access and privacy issues, participants also emphasized the importance of focusing on the user experience to ensure the portal is useful. Participants noted that registration processes needed to be simplified and streamlined to encourage portal use:

Making our consent form electronic. Instead of having to come in and sign a piece of paper, that process is now online. You can sign up for a patient portal account through an electronic form. You can upload a picture of your driver’s license, and that has made all the difference in helping people get enrolled with a patient portal account.

Participant #167, chief medical information officer

However, many of the barriers to streamlined enrollment were related to identity verification to ensure parents were not registering for their adolescent’s account. Furthermore, some participants described the need to engage adolescent end users to ensure the interfaces are user-friendly:

In general, I’m not sure if people have set about to do studies from the patient perspective, on how difficult or easy it is to use any of these personal health records or the portals that they have, so there’s a lot of improvement that could be done in terms of making these user-friendly.

Participant #116, chief medical information officer

#### Collaborating and Advocating

##### Engage Key Stakeholders Within the Institution

When developing policies, participants stressed the importance of engaging multiple stakeholders within the health care institution, clinical teams, and families to ensure the policies were responsive to the needs of these parties and as broadly acceptable as possible: “Communication, communication, communication, get everybody involved early and speak to all the people who were involved” (participant #121, clinical informaticist). Stakeholders included teens, parents, legal and compliance teams, clinicians, informaticists, information technology support staff, and other frontline staff involved in registration and enrollment. Participants advised multiple approaches to engaging families, including advisory boards, open forums, and satisfaction surveys:

If you don’t have a family advisory board or a teen advisory board, that is really key. Then I also think just having open forums to hear what people say because we’re not perfect.

Participant #167, chief medical information officer

Yet, some participants felt that the adolescent voice was lacking at their institution:

I don’t think there’s any adolescent voices being represented. I think there’s a lot of parental voices being represented, but I don't think in our situation, I don’t think that there’s any—there’s ever been a teen at the table in adolescent practices even in creating clinic culture.

Participant #144, clinician

Within the clinical team, participants advised administrators to consider differences in practice patterns and patient populations when developing and implementing policies:

We had to have a working group with legal, with experts in adolescent care, and really with care providers from different venues. Outpatient versus ED, versus urgent care, versus inpatient are all very different sets of episodes of care, and information types. The needs and perspectives, the providers are also gonna be different.

Participant #153, chief medical information officer

One participant described the need to continue tracking the expected and unexpected outcomes of policies after implementation:

Put this on your agenda regularly. How are we accomplishing this, and what are our gaps? For our organization, I feel like we—and how are we gonna continually assess it? We are not successfully doing that.

Participant #163, chief medical information officer

Another important aspect was collaborating with hospital administration to understand organizational priorities to most effectively advocate for adolescents:

Know what your state laws are but also know what are your guiding principles as an organization with respect to adolescent health. Those might be in conflict. Then you have to determine what is your risk tolerance when it comes to that.

Participant #20, chief medical information officer

##### Collaborate With Colleagues at Other Institutions

Given the multiple challenges inherent in developing and implementing portal policies for adolescents, participants emphasized the importance of collaborating and sharing best practices with colleagues. Additionally, some participants noted how institutions within the same state are implementing very different policies. As such, some participants called for institutions within states to strive toward consensus on a common approach, even though consensus would be difficult to reach:

I would say that to the extent you can within your state, come together across institutions and try to at least discuss a common approach...I think some uniformity agreements which is straight in will never get there, but it’s great to strive for.

Participant #180, chief medical information officer

Furthermore, institutions should share their best practices with other institutions:

Then I would encourage institutions to share best practice. If something’s working put it out there so that other people that are using the same EHR can see what you’re doing and learn from it as well.

Participant #37, clinical informaticist

Finally, a participant from an integrated health system advised informatics leaders from major academic pediatric hospitals to consider smaller pediatric centers with fewer resources when recommending standards and policies:

The big pediatric institutions in the country, I would ask that they really think about where and how a lot of pediatric care is delivered in the country...How do we help the great work that’s happening at some of the big, pediatric centers from that standpoint really get into these other places in the country that are providing lots of pediatric care?

Participant #36, clinical informaticist

##### Advocate With External Parties for Adolescent and Pediatric Issues

Participants described the need to pressure EHR vendors to develop necessary technical functions in the EHR, especially related to granular differential sharing of content between adolescent and proxy portals. Currently, each health system has to modify its EHR instance to meet these unique sharing needs, and the capacity to differentially share information between proxy and adolescent portals is limited:

I think the other thing is to continue to pressure the vendors to make this easier to do out of the box, and that’s really where the CEOs have the ear of the leads of the vendor, EHR vendors, and so really to push that this is something that needs to be really addressed at the vendor level. It’s crazy for us all to be doing our own build on this.

Participant #155, chief medical information officer

Additionally, participants described the need to advocate and lobby legislators to improve laws and regulations by adding specificity around the type of sharing required, age of adolescence, and parental and adolescent rights: “Encourage Uncle Sam [United States Government] to write rules that make sense specific to the pediatric population” (participant #109, chief medical information officer). One participant described the importance of engaging with legal counsel that was external to the hospital, to avoid being “stuck in an institutional echo chamber” (participant #158, clinical informaticist). To support these advocacy efforts, 1 participant called for guidance from national organizations:

It would be really great if one of our professional organizations would come forward, like the [American Academy of Pediatrics] and say like, “This is what we believe,” in the context of information blocking and the Cures Act...If you could refer to some external expert body...I think it would really lend that extra weight.

Participant #90, chief medical information officer

## Discussion

Informatics administrators described guiding principles that aimed to maximize transparency while complying with laws, respecting parental roles, protecting the adolescent’s health and safety, and ensuring that the portal remains a useful tool. These overarching guiding principles align well with prior policy statements from professional organizations, providing an evidence base to support these statements. For example, the American Academy of Pediatrics advised health care institutions to ensure medical teams are “aware of state and federal requirements and to assist them in complying with standards, rules, and regulations” [23]. The Society for Adolescent Health and Medicine described the crucial importance of institutions determining which information will be shared with patients and proxies, as well as ensuring this information sharing complies with the 21st Century Cures Act Final Rule. This organization specifically recommended that clinicians and institutions know and abide by state and federal laws and advocate on behalf of the adolescent with key stakeholders within and outside of the institution [24]. The American College of Obstetrics and Gynecology similarly called for awareness and compliance with pertinent laws, while ensuring adolescents have the ability to have private, confidential communication with their obstetrician-gynecologists. Additionally, they advised clinicians to be aware of their institution’s policies and capabilities regarding confidentiality when they are documenting sensitive information [14].

While we observed general agreement on many of these overarching principles, these goals can be conflicting when put into practice. For many adolescents with chronic illness, for example, providing parents with information is essential to support that child’s complex care needs. Yet, technological limitations and interpretations of state laws led many institutions to limit parental access to information that is essential to support the adolescent [16]. Furthermore, the usefulness of portals is greatly diminished when institutions limit available information. For example, we previously found that some institutions shut down the portal completely during adolescence for parents and adolescents, and other institutions only provide minimal information such as vaccination status and vital signs [16]. Contrarily, other adolescents might need information withheld from their parents to protect them from abuse or harm. Inadvertent disclosure of sensitive information can subvert the adolescent’s right to privacy, diminish trust in clinicians, and decrease the adolescent’s transparent engagement with the health care system [6,8,9,30]. Furthermore, some adolescents might forgo sensitive care (ie, sexually transmitted infections, pregnancy, and drug abuse) if they are not guaranteed confidentiality. Some participants described how adolescents should be empowered to decide on this balance between confidentiality and usefulness by determining how much access they will permit their parents. Yet, some institutions considered this practice to be in conflict with their state’s laws.

While it is important to ensure adolescent’s confidentiality, the role of parents in supporting adolescents must not be ignored. Most adolescents rely on their parents for medical management, insurance and financial support, transportation, assistance in decision-making, emotional support, and consent to treatment [11]. Furthermore, some adolescents have limited interest in using portals, scheduling appointments, filling prescriptions, and managing other aspects of care. For adolescents with serious or debilitating illness, this reliance on parents can be even greater. Depending on each adolescent’s unique situation, protecting privacy can either be essential to providing safe and effective health care or a major barrier to health and safety. When developing policies, the beneficial role of parental involvement must be weighed against the potential harms of inadvertent disclosure. To the extent possible, administrators should leverage available technology to minimize these disclosures while also allowing adolescents to involve parents in their health care to the extent desired or required by law.

These data highlight several targets for ongoing advocacy efforts, further supporting prior calls for advocacy in this area [24]. Within each institution, pediatricians can advocate with institutional leaders to ensure policies are informed by the adolescent’s best interests and the voices of key stakeholders. To address technological limitations, institutions can advocate with EHR companies to develop tools and workflows to permit differential sharing of information to the adolescent and proxy. Pediatricians and pediatric institutions can also advocate with lawmakers at the state and federal levels to support legislation that is informed by the experiences of adolescents, parents, and clinicians. Future studies should aim to capture the perspectives of adolescents and parents to better inform these advocacy efforts. To strengthen these advocacy efforts, health care institutions within and across states should attempt to align policies and priorities to the extent possible. While many participants described myriad challenges to gaining a national consensus, intrastate consensus should be more feasible, since all institutions are responding to the same state laws.

This study has limitations that should be considered. We limited enrollment to hospitals with at least 50 dedicated pediatric beds, which could underrepresent the challenges of hospitals in integrated health systems with a smaller pediatric presence. Our results could be biased toward larger pediatric hospitals, which could limit the representativeness of our data. Also, participants could have moderated their responses during interviews due to social desirability bias. Furthermore, we did not design this study to evaluate specific characteristics of different EHR platforms, which could have provided additional practical information.

Informatics administrators provided guiding principles for adolescent portal access policies that aimed to balance the competing needs of adolescent confidentiality and the usefulness of the portal. As bedrock principles, these policies must prioritize the adolescent’s health and safety while complying with state and federal laws. The main limiting factors in balancing these priorities were technological limitations and institutional interpretations of laws. Although most participants agreed on broad principles, we observed disagreements about how to specify the principles into policies. Institutions and clinicians should strive for consensus on principles to strengthen advocacy efforts with institutional leadership, EHR vendors, and lawmakers.

## Appendix

Multimedia Appendix 1 Standards for Reporting Qualitative Research (SRQR) checklist.

Multimedia Appendix 2 Interview guide.

## Abbreviations

CHA: Children’s Hospital Association
EHR: electronic health record
SRQR: Standards for Reporting Qualitative Research
