# Supplementary material for: Guiding Principles for Adolescent Web-Based Portal Access Policies: Interviews With Informatics Administrators
Source: JMIR Pediatr Parent. 2024 Mar 11;7:e49177. doi: 10.2196/49177 (PMC10964143; doi:10.2196/49177)
Supplement: Multimedia Appendix 2 [file pediatrics_v7i1e49177_app2.docx]

**Multimedia Appendix 2.** Interview Guide

**[Introduction]**

Hello, may I speak with ___________? This is __________, and I’m calling from Washington University in St. Louis. Is now still a good time for the interview? Great, thank you. Have you had a chance to review the consent document from the previous email?

**[If no: “Do you have access to your email to review that form now?” If not – “Okay, I can read the consent document to you over the telephone.”]**

Do you agree to be part of this study?

**[PRESS RECORD]**

Great. Thank you. First, I thought I would tell you a little about what we’ll talk about today. As you know, the 21^st^ Century Cures Act mandates that healthcare systems provide easy access to electronic health information. Children’s hospitals are taking different approaches to this access in adolescent care. We are interviewing informatics experts who are overseeing this access to better understand how different institutions are implementing this access, and how they are promoting adolescent engagement. I have 5 main questions to ask today. Please feel free to ask for any clarification that might be helpful as we move through the questions. This whole interview should take about 15 to 20 minutes. Also, we will refer to parents in these questions, but we mean parents and legal guardians.

Before we get started, I have three quick questions.

1. What is your professional role in informatics?
2. What EHR do you use in your pediatric hospital.
3. Was your instance of the EHR built specific for pediatric use, or for both adult and pediatric use?
4. Do you have a pediatric focused (or pediatric dedicated) informatics team, in addition to your adult team?

Okay, now I will start with the first question.

1. **First Question - What are your institution’s current policies for providing portal access to adolescents and their parents or guardians?**
   1. Are adolescents permitted access?

**(If yes)**

- - 1. Are all adolescent accounts the same? Do you have tiered accounts for different types of access (level of access)?
    2. Does your organization require parental consent for adolescents to access their information?
    3. At what age do you provide EHR access to adolescent patients?

**(If no)**

- - 1. How did your organization come to this decision?
  1. Are parents permitted proxy access for adolescent patients?

**(If yes)**

- - 1. Is parental access different for parents of young children versus parents of adolescents? How so?
       1. At what age does this level of access for parents/proxies change?
    2. Does your organization require adolescent assent for parental proxy access?

**(If no)**

- - 1. How did your organization come to this decision?
  1. What have been the biggest challenges in designing and implementing these EHR policies for adolescents and their parents?
  2. Are you happy with the level of access your institution provides?

1. **Second Question - What steps has your institution taken to engage adolescents in accessing their portal?**
   1. Are there specific clinics that have developed projects or best practices related to engaging adolescents? (How so?)
   2. Are there clinics that are more hesitant to engage adolescents in accessing their portals? (How so?)
   3. Are there specific clinics that have greater challenges or dificulties in engaging adolescents? (How so?)
2. **Third Question - What feedback have you received from adolescent patients and their families about their ability to access EHI through the portal?**
   1. (If only negative) What about any positive feedback?
   2. (If only positive) What about any negative feedback?
3. **Fourth Question - In a perfect world, how would we ideally engage adolescents and parents in using their patient portals?**
   1. What makes this ideal?
      1. What should be the guiding principles when institutions design proxy portal access for parents of adolescents? [OK to skip if short on time]
      2. What should be the guiding principles when institutions design portal access for adolescents? [OK to skip if short on time]
   2. What are the challenges to reaching this ideal state?
4. **Last Question - What important lessons have you learned since the 21^st^ Century Cures Act went into effect?**
   1. What could have gone better at your organization? [OK to skip if short on time]
   2. What advice do you have for administrators at other institutions about providing EHI access through portals in adolescent care?

**[Closing]** Anything else that you think I should know about providing EHR access in adolescent care? [OK to skip if short on time]
